# Cancer-Associated Fibroblasts in Hepatocellular Carcinoma and Cholangiocarcinoma

**DOI:** 10.1016/j.jcmgh.2023.01.006

**Published:** 2023-01-25

**Authors:** Fan Ying, Mandy Sze Man Chan, Terence Kin Wah Lee

**Affiliations:** 1 Department of Applied Biology and Chemical Technology, The Hong Kong Polytechnic University, Hong Kong; 2 State Key Laboratory of Chemical Biology and Drug Discovery, The Hong Kong Polytechnic University, Hong Kong

**Keywords:** Primary Liver Cancer, Hepatocellular Carcinoma, Cancer-Associated Fibroblasts, Cholangiocarcinoma, Origin, Immune Suppression

## Abstract

Primary liver cancer (PLC) includes hepatocellular carcinoma and intrahepatic cholangiocarcinoma and is the sixth most common cancer worldwide with poor prognosis. PLC is characterized by an abundant stromal reaction in which cancer-associated fibroblasts (CAFs) are one of the major stromal components. Solid evidence has demonstrated the crucial role of CAFs in tumor progression, and CAF abundance is often correlated with poor clinical outcomes. Although CAFs are regarded as an attractive and promising target for PLC treatment, a poor understanding of CAF origins and heterogeneity and a lack of specific CAF markers are the major hurdles to efficient CAF-specific therapy. In this review, we examine recent advances in the understanding of CAF diversity in the context of biomarkers, subtypes, and functions in PLC. The regulatory roles of CAFs in extracellular matrix remodeling, metastasis, cancer stemness, and therapeutic resistance are summarized. With an increasing link between CAF abundance and reduced antitumor immune responses, we provide updated knowledge on the crosstalk between CAFs and immune cells within the tumor microenvironment, which leads to immune resistance. In addition, we present current CAF-targeted therapies and describe some future perspectives. A better understanding of CAF biology will shed light on a novel therapeutic strategy against PLC.

**Summary**

Accumulating evidence provides the critical roles of cancer-associated fibroblasts in extracellular matrix remodeling, metastasis, cancer stemness, immune modulation, and therapeutic resistance. A better understanding of cancer-associated fibroblast biology will shed light on a novel therapeutic strategy against primary liver cancer.

Primary liver cancer (PLC) ranks sixth in cancer incidence and third worldwide as a leading cause of cancer-related death, with an estimated incidence of more than 1 million cases by 2025.^1^ Compared with other malignancies, hepatocellular carcinoma (HCC) with cholangiocarcinoma (CCA) are by far the second-most common, accounting for approximately 70% and 15% of cases, respectively, and represents both ends of the spectrum of primary malignant tumors.^2^

HCC is derived from the malignant transformation of hepatocytes. Chronic hepatitis B virus and hepatitis C virus infections are considered the most prominent risk factors for HCC development, accounting for more than half of cases.^3^ Additionally, alcohol intake or chronic inflammatory conditions, such as nonalcoholic steatohepatitis (NASH) associated with metabolic syndrome or diabetes mellitus, are becoming the fastest growing causes of HCC, particularly in western countries. Reports on mutational signatures have also demonstrated that aristolochic acid and tobacco may be potential pathogenetic factors associated with HCC.^4^ Because of the delayed diagnostic time of HCC, most patients are discovered and considered as inoperable.^5^

CCA is thought to originate from the malignant transformation of cholangiocytes and is the most aggressive malignant tumor of the biliary tract. CCA is generally classified as intrahepatic and extrahepatic.^6^ Among these, intrahepatic cholangiocarcinoma (iCCA) together with HCC is considered one of the most common PLC. Similar to HCC, CCA also develops from hepatitis B and C virus infection and unresolved inflammatory conditions, including obesity, diabetic mellitus, and congenital hepatic fibrosis. Specifically, the main predisposing condition for CCA in western countries is primary sclerosing cholangitis.^7^ Notably, in Southeast Asia, liver fluke infections have the highest correlated risk of CCA, which spreads to the bile ducts, gallbladder, and liver parenchyma and causes chronic infection and inflammation.^8^ Additionally, intrahepatic stones could also be one of the major causes of iCCA.

For patients with HCC receiving curative-intent treatments, the 5-year overall survival rate is more than 50%, whereas for patients with iCCA receiving curative-intent surgery, the 5-year overall survival rate is only approximately 5%–17%, suggesting that patients with iCCA might have a worse prognosis than those with HCC. Locoregional therapies could be an alternative option for patients who cannot receive surgical intervention. Systemic therapy is also recommended for advanced liver cancer.^9^ However, in combination with the high degree of malignancy and the severity of neighboring invasion, the optimal management of HCC and CCA remains an extreme challenge and is long in duration.

## The Cellular Origins of Cancer-Associated Fibroblasts in Hepatocellular Carcinoma and Cholangiocarcinoma

**Figure 1:**
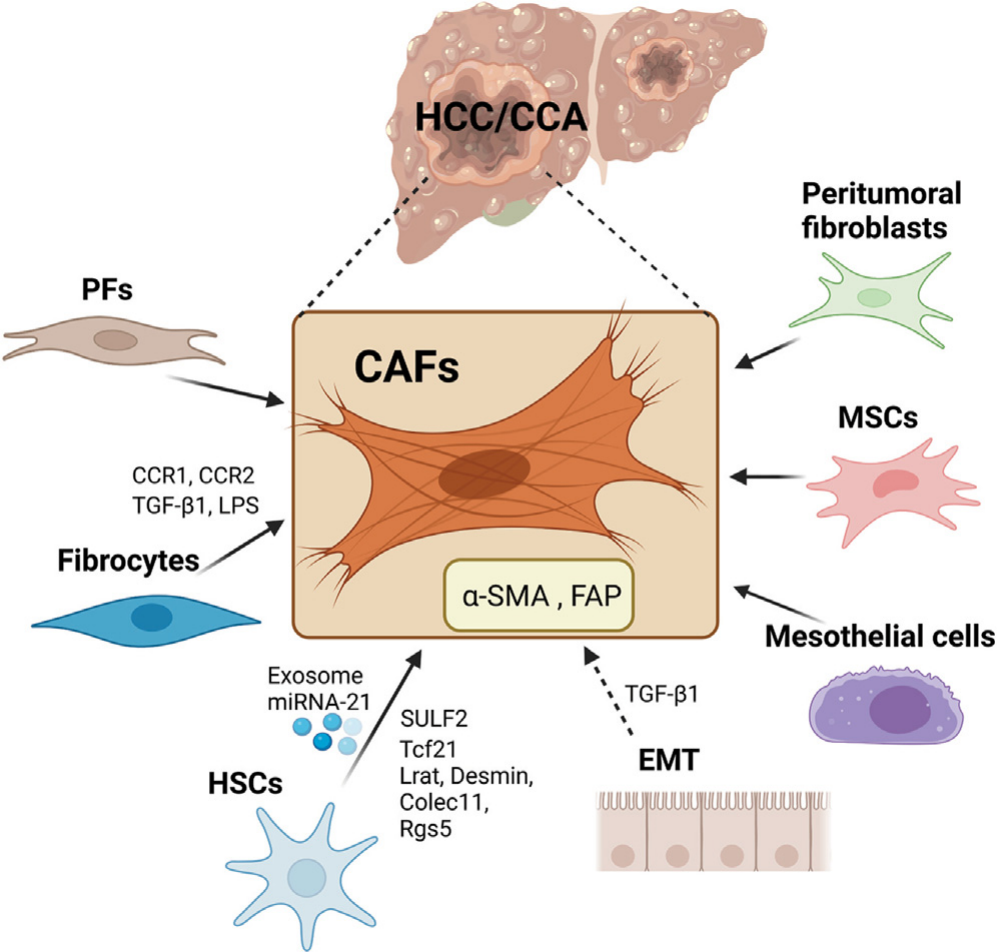
**Overview of the cellular origins of CAFs in HCC and CCA.** CAFs can originate from multiple cell types including HSCs, PFs, fibrocytes, EMT, mesothelial cells, MSCs, and peritumoral fibroblasts. Several factors and exosome miRNA were reported as the drivers of those transdifferentiations.

During hepatocarcinogenesis, persistent liver damage and advanced stage of liver fibrosis are key drivers. To be noted, more than 90% of HCC cases develop from a background of liver cirrhosis, and approximately one-third of patients with liver cirrhosis ultimately develop HCC.^10^ Indeed, an increasing number of studies suggest that the reconstruction of the tumor microenvironment (TME), a suitable environment for tumor cells, promotes tumor progression.^11^ In HCC, the reciprocal crosstalk among the TME, which includes cancer-associated fibroblasts (CAFs), immune cells, endothelial cells, extracellular matrix (ECM) elements, and HCC cells, significantly reinforces proliferation, migration, metastasis, and chemoresistance.^11^ CAFs, also called myofibroblasts, are a heterogeneous group of activated fibroblasts in the tumor stroma. Hepatic CAFs, commonly marked with smooth muscle alpha-actin (α-SMA) and fibroblast activation protein alpha (FAP) expression, are the main sources of collagen-producing cells, which is one of the critical and abundant components in the TME, and have been implicated in the progression of HCC.^12^ According to the studies, the origins of CAFs in HCC and CCA could be from a variety of cell types (Figure 1).

### Hepatic Stellate Cells

Activated hepatic stellate cells (HSCs) have been reported as the predominant origin of hepatic myofibroblasts in HCC.^13^ HSCs are liver-specific mesenchymal cells that maintain interactions with sinusoidal endothelial cells and hepatic epithelial cells.^14^ During chronic liver injury, HSCs downregulate vitamin A–constraining lipid droplets and neural markers; migrate to the pericentral areas; and transdifferentiate into proliferative, fibrogenic, myofibroblasts.^15^ Fate-tracing revealed that HSCs gave rise to more than 80% of collagen-producing myofibroblasts in carbon tetrachloride (CCl_4_)-induced toxic liver fibrosis and cholestatic-induced biliary liver fibrosis.^16^ Zhou et al^17^ showed that HCC cells release exosomal miRNA-21, which mediates the differentiation of HSCs into α-SMA and FAP-positive CAFs, and activated CAFs express high levels of angiogenic cytokines that further promote HCC progression in subcutaneously transplanted tumors. Wang et al^18^ cocultured HSCs with HCC cells and revealed that sulfatase 2 secreted by HCC cells promoted HSC-CAF differentiation, which was associated with the upregulation of the CAF markers tenascin-C and stromal cell-derived factor 1 (SDF1) and promoted epithelial-to-mesenchymal transition (EMT) in HCC cells, and eventually led to HCC development. According to The Cancer Genome Atlas database, a 12-marker panel of CAFs in HCC has been identified. When the HSC cell line LX2 was cocultured with conditioned medium (CM) from the HCC cell line Huh7, LX2 cells showed high levels of 12 marker proteins in additional to α-SMA and FAP expression.^19^ By performing single-cell RNA sequencing (scRNA-seq), Wang et al^20^ identified that transcriptional factor 21 was preferentially expressed by periportal and pericentral HSCs that generated the most CAFs in a mouse liver fibrosis model, supporting the functional contribution of HSC-associated liver fibrosis to the progression of HCC. Tracing in combination with single-cell transcriptomics was used, Bhattacharjee et al^21^ illustrated that most CAFs showed abundant expression of the HSC signature, suggesting that HSCs are the primary source of liver metastasis–associated CAFs. Interestingly, in an iCCA model, HSCs have been also confirmed to be the main source of CAFs, and HSC-derived CAFs are the dominant tumor-interacting populations associated with iCCA progression.^22^ Analysis of scRNA-seq CAF data showed that most CAFs expressed an HSC signature, including lecithin retinol acyltransferase, Desmin, collectin subfamily member 11, and regulator of G protein signaling 5, suggesting an HSC origin, whereas less than 10% expressed the markers of portal fibroblasts (PFs), another fibrogenic population in iCCA. Additionally, scRNA-seq data of human and murine iCCA suggested that HSCs-CAFs represented the subpopulation with the most ligand-receptor interactions with tumor cells, and were associated with decreased survival and increased recurrence risk in iCCA.^22^

### Portal Fibroblasts

PFs are located in the periportal area and maintain the integrity of the biliary tree and portal tract.^23^ PFs can be distinguished from HSCs by expressing of Thy1, fibulin (Fbln) 2, elastin (Eln), gremlin1, mesothelin, mucin 16, and ecto-ATPase nucleoside triphosphate diphosphohydrolase-2. Activated PFs with high expression of Thy1 and CD34 contributed to the development of hepatic myofibroblasts during the early progression of cholestatic fibrosis, although HSCs give rise to the major sources of CAFs during the late stages of cholestatic liver injury and during hepatotoxic liver injury.^24^ Yang et al^25^ performed single-cell and lineage-tracing analysis and revealed that PFs primarily contributed to collagen-producing myofibroblasts in BDL-liver, which was associated with the expression of PF-specific markers including Thy1, Fbln1, Eln, dermatopontin, microfibril-associated glycoprotein 4, and growth arrest specific 6. The number of PFs was also dramatically elevated in the bile duct ligation-induced liver fibrosis.^25^ A subset of PFs with mesenchymal stem cell features (PMSCs) was initially discovered by using scRNA-seq and could generate myofibroblasts.^26^ Researchers built oligogene signatures and found that slit homolog 2 (SLIT2) was a prototypical gene of the PMSCs signature, which promoted HSC survival and largely expanded with hepatic fibrosis progression.^26^ Itou et al^27^ characterized CAFs in the metastatic lymph nodes and showed that PFs were the sources of CAFs at primary sites associated with positive expression of platelet-derived growth factor receptor-β (PDGFR-β), FBLN2, and Thy1 in human iCCA.

### Fibrocytes

Fibrocytes originate from hematopoietic stem cells and are characterized by the expression of fibroblast markers (collagen type I, vimentin, and fibronectin) and hematopoietic cell markers (cluster of differentiation [CD] 45, CD34, CD11b, CD54, CD80, CD86; major histocompatibility complex [MHC] II; Ly-6G/Ly-6C; C-C motif chemokine receptor [CCR] 2, CCR1, CCR7, and CCR5).^28^ Fibrocytes were first described as “CD45 and collagen type I expressing leukocytes that mediate tissue repair and are capable of antigen presentation to naive T cells.”^29^ Studies have suggested that in response to liver injury, fibrocyte migration from bone marrow was detected in both the liver and spleen, and these cells gave rise to myofibroblasts, which were regulated by the CCR2 and CCR1. In response to CCL4-damaged liver, bone marrow–derived fibrocyte was also regulated by transforming growth factor-beta 1 (TGF-β1) and the intestinal permeability marker lipopolysaccharide (LPS), indicating that the release of TGF-β1 and LPS plays an important role in fibrocyte trafficking.^30^

### Epithelial-to-Mesenchymal Transition

EMT is a process in which endothelial cells lose their characteristic markers, acquire a migratory phenotype, and gain enhanced levels of mesenchymal markers.^31^ A growing body of research has suggested that CAFs with high aggressiveness originate from HCC cells undergoing EMT. Zeisberg et al^32^ performed lineage-tracing experiments and demonstrated that hepatocytes undergoing EMT may contribute to the population of FSP-1 (fibroblast specific protein-1)-positive cells in CCL4-induced liver fibrosis. Consistently, Zou et al^33^ demonstrated that in patients with HCC, hypoxia correlated with increased expression of FAP, a typical CAF marker. In addition, increased FAP in HCC cell lines was parallel with EMT markers, including E-cadherin, Snail, and Twist-related protein. In another study, TGF-β, which promotes EMT and stimulates fibroblast maturation, has been demonstrated to induce α-SMA-mediated CAF differentiation and contribute to HCC invasion and metastasis.^34^^,^^35^ However, the contribution of EMT to fibrogenic CAFs remains controversial. Cell fate mapping experiments in mice suggested that hepatocytes and cholangiocytes do not undergo EMT during liver fibrosis and do not give rise to the generation of myofibroblasts or the markers of myofibroblasts.^36^^,^^37^ In a rat CCA model, CAFs in CCA do not originate from hematopoietic stem cells or bone marrow–derived fibrocytes, and whether they originate from endothelial cells through EMT remains unknown.^38^ Therefore, more studies are required to clarify this issue.

### Others

In addition to these possible cell sources, mesothelial cells, mesenchymal stromal cells (MSCs), and peritumoral fibroblasts can also be transformed into CAFs. Based on the findings by Asahina, mesothelial cells undergo HSC and myofibroblast transdifferentiation during CCL4-induced liver fibrogenesis, whereas during cholestatic liver damage, mesothelial cells only give rise to HSCs but not myofibroblasts.^39^^,^^40^ Multipotent MSCs migrate to the area of liver fibrosis and the HCC microenvironment, and can influence tumor initiation and progression.^41^ A study revealed that after being cocultured with HCC cell line, MSCs exhibited the properties of CAFs and were associated with upregulated tenascin-C and chemokine (C-X-C motif) ligand (CXCL) 12.^42^ Another recent study suggested that human adipose MSCs had significantly increased the expression of CAF markers, including α-SMA, vimentin, c-MYC, matrix metalloproteinases (MMP) 2, vascular endothelial growth factor, fibroblast growth factor receptor1 (FGFR1), interleukin (IL)-6, IL-8, and Tenascin-C, when cocultured with HCC cells, thereby acquiring a CAF-like phenotype.^43^ Apart from that, peritumoral fibroblasts can be transdifferentiated into CAFs induced by lysophosphatidic acid (LPA), which is released from HCC cells.^44^

## Biomarkers of Cancer-Associated Fibroblasts in Hepatocellular Carcinoma and Cholangiocarcinoma

Because the expression of CAF markers is extremely heterogeneous and varies strongly between different CAF subpopulations in HCC and CCA tissues, identifying unique CAF biomarkers remains challenging. Although a large number of CAF biomarkers have been reported, such as a-SMA, Thy, FAP, vimentin, and FSP-1,^18^^,^^19^^,^^22^^,^^43^ none of them are specific to CAFs, because they overlap with distinct subtypes. The present review summarizes CAF markers based on different subpopulations, as shown in Table 1.^45^, ^46^, ^47^, ^48^, ^49^, ^50^, ^51^, ^52^, ^53^, ^54^, ^55^, ^56^, ^57^, ^58^, ^59^, ^60^, ^61^, ^62^, ^63^, ^64^

**Table 1:** CAF Signature/Biomarkers in HCC and CCA

CAF origin	Signature/biomarkers	References
HSCs	α-SMA, FAP	17
	VEGF, MMP2, MMP9, bFGF, TGF-β	17
	Tenascin-C, SDF1	18
	CXCL5, IGFL1, IGFL2, MMP1, ADAM32, ADAM18, FGF5, FGF8, FGF17, FGF19, FGF4, FGF23	19
	Tcf21	20
	Lrat, Desmin, Colec11, Rgs5	22
	Positive: Lrat, Reln, Rgs5; Negative: Acta2, Col1a1, Col8a1, Col15a1, Crlf1 Fbn2, Desmin, GFAP, Glialfibrillary acidic protein	22
	VEGF, MMP9	45
	Thy1, α-SMA, FAP	46
	LSD1	47
	Desmin, GFAP, Glialfibrillary acidic protein	48
PFs	Msln, Upk1b, Upk3b, Gpm6a	22
	Thy1, CD34	24
	Thy1, Fbln1, Eln, Dpt, Mfap4, Gas6	25
	SLIT2	26
	PDGFR-β, fibulin-2, Thy-1	27
	Elastin, Thy1, Ntpdase 2	49
Fibrocytes	Collagen type I, Vimentin, fibronectin, CD45, CD34, MHCII, CD11b, Gr-1, CD54, CD80, CD86, CCR2, CCR1, CCR7, CCR5	28
HCC cells	α-SMA, FAP	17
	FAP	19
	FSP-1	32
	ITGA2, ITGAV, ITGA1	50
	CXCL11	51
ICC cells	CD146, IL-6, EZH2	52
	α-SMA, FSP-1, PDGFRβ	53
	PDGFD	54
Mesothelial cells	Msln, Upk1b, Upk3b, Gpm6a	22
	Gpm6a	39
	Msx2	55
	Wilms	56
	ITGA8	57
Mesenchymal stromal cells	Tenascin-C, CXCL12	42
	α-SMA, Vimentin, c-MYC, MMP2, VEGF, IL-6, FGFR1, IL-8, Tenascin-C	43
	Positive: CD73, CD90, CD105, CD44, CD13, CD29, CD166 Negative: CD31, CD34, CD45, CD117, HLA-DR	58
HCC-associated CAFs	COL1A1, COL4A1, COL6a2	50
	Versican	59
	ACTA2, COL1a1	60
CCA-associated CAFs	Podoplanin	61, 62
	CD10	63
	Periostin	64

Acta2, actin alpha 2; ADAM, a disintegrin and metallopeptidase domain; bFGF, basic fibroblast growth factor; CAF, cancer-associated fibroblasts; CCA, cholangiocarcinoma; Colec11, collectin subfamily member 11; Crlf1, cytokine receptor like factor 1; Dpt, dermatopontin; EZH2, enhancer of zeste 2 polycomb repressive complex 2 subunit; Fbn, fibrillin; FSP-1, fibroblast-specific protein 1; Gas6, growth arrest specific 6; GFAP, glial fibrillary acidic protein; Gpm6a, glycoprotein m6a; HCC, hepatocellular carcinoma; HSC, hepatic stellate cells; ICC, intrahepatic cholangiocarcinoma; IGFL, insulin growth factor-like family member; ITGA, integrin subunit alpha; Lrat, lecithin retinol acyltransferase; LSD1, lysine-specific demethylase 1; Mfap4, microfibril-associated glycoprotein 4; MHC, major histocompatibility complex; Msln, mesothelin; Msx2, msh homeobox 2; PDGFR, platelet-derived growth factor receptor; PF, portal fibroblasts; Rgs5, regulator of G protein signaling 5; SDF1, stromal cell-derived factor 1; Tcf21, transcriptional factor 21; TGF-β1, transforming growth factor-beta 1; Upk, uroplakin; VEGF, vascular endothelial growth factor.

Recently, scRNA-seq has provided a wealth of data on CAF biomarkers in HCC and CCA. Chiavarina et al^65^ used proteomics and scRNA-seq analysis and unveiled 3 major CAF subpopulations in human HCC that expressed biomarkers including COL1A1, Thy1, RGS5, and NDUFA4 mitochondrial complex associated like 2 (NDUFA4L2) (HSC markers, CAF_HSC), PDGFRA, and MMP23B (PF markers, CAF_Port), and myosin heavy chain 11 (MYH11) and calponin 1 (vascular smooth muscle markers, CAF_VSMC). Specifically, their team showed that prolargin was a novel biomarker in the subpopulation of PF-derived CAFs and that CAF-derived prolargin interferes with interaction of tumor cells with endothelial cells, which suppresses tumor angiogenesis.^65^ Meng et al^50^ assessed the scRNA-seq data of patients with HCC and observed a significant distinction among CAFs associated with differential genes COL1A1, COL4A1, and COL6A2, which are enriched in the ECM-receptor interaction pathway. Further analysis revealed that COL1A1 and ITGA2 have the highest interaction mediated by crosstalk between CAFs and tumor cells.^50^ According to the analysis of scRNA-seq in human iCCA, another recent study discovered 6 distinct fibroblast subpopulations including vascular CAFs defined by CD146, MYH11, gap junction protein alpha 4, and RGS5 expression and upregulated IL-6 expression; matrix CAFs defined by collagen molecules (COL5A1, COL5A2, and COL6A3), periostin, fibronectin 1, lumican, decorin, and versican expression; inflammatory CAFs defined by FBLN1, IGF1, CXCL1, IGF-binding protein 6, secretory leukocyte peptidase inhibitor, serum amyloid a1 expression and associated with inflammatory response regulation and complement activation; antigen-presenting CAFs defined by CD74, HLA-DRA, and HLA-DRB1 expression; EMT-like CAFs defined by the epithelium-specific marker genes keratin (KRT) 19, KRT8, and serum amyloid a1; and lipofibroblasts defined by lipid metabolism and processing-related genes, such as apolipoprotein a2, fatty acid binding protein (FABP) 1, FABP4, and frizzled related protein.^52^

In summary, the expression of CAF biomarkers is very heterogeneous and mainly depends on the CAF subtypes analyzed in these studies. Thus, investigations of CAF-specific markers are still vital.

## The Role of Cancer-Associated Fibroblasts in Extracellular Matrix Modelling

ECM is a collection of proteins that provide critical signals to preserve tissue scaffold structure and organ homeostasis, and regulate cell growth and apoptosis. During cancer progression, alterations in ECM drive tumor cell initiation, invasion, metastasis, and resistance.^66^ Many solid tumors, such as HCC and CCA, express large amounts of ECM components, including fibrillar collagen, laminin, proteoglycans, and fibronectin, which mostly come from activated CAFs.^67^ Santamato et al^68^ reported that activated HSC could produce and secrete the ECM component laminin-5, which contributes the migration and invasion of HCC cells via the mitogen-activated protein kinase ERK kinase/extracellular-signal-regulated kinase pathway. Increased and reorganized ECM protein secreted by CAFs reinforce the deposition of fibrillar collagen, thereby mechanically giving rise to ECM stiffening and promoting tumor cell proliferation and invasion.^66^ Schrader et al^69^ used an in vitro system of “mechanically tenable” matrix-coated polyacrylamide gels and revealed that enhanced matrix stiffness promoted proliferation and chemotherapeutic resistance of HCC cells via hepatocyte growth factor (HGF)-induced signaling responses. Interestingly, Olsen et al^70^ demonstrated that mechanically stiff substrates (coated with different ECM proteins or poly-L-lysine) were required by primary rat HSCs to differentiate into myofibroblasts, indicating that liver stiffness-dependent HSC differentiation required matrix proteins and the generation of mechanical tension, which is probably a key factor driving the progression of liver fibrosis. Abundant studies have confirmed that liver stiffness is closely correlated with the risk of developing HCC in patients, and its assessment can serve as a potential predictor of HCC development.^71^, ^72^, ^73^ In addition, MMPs are key mediators of ECM degradation.^74^ MMP9 was reported to induce EMT in human kidney fibrosis.^75^ Of interest, coculturing activated primary rat HSCs with type I collagen could stimulate the ECM remodeling properties of HSCs by inducing MMP9 expression.^76^ Furthermore, reconstruction of the fibrous structure by adjusting the alignment of collagen fiber is an alternative mechanism in ECM remodeling. Jung et al^77^ found that high forces generated by premetastatic cancer cells have the ability to align ECM fibrils and therefore enhance the delivery of CAF-promoting factors toward the stroma, resulting in the activation of myofibroblasts. Moreover, the contractility of CAFs is considered as another physical mechanism that remodels the ECM by generating mechanical forces by widening the pores of the ECM or aligning collagen fibers, contributing to directional invasion of tumor cells.^78^ However, whether this is true in HCC or CCA models is still unknown.

## The Role of Cancer-Associated Fibroblasts in the Regulation of Metastasis

Accumulating evidence has shown that CAFs control tumor growth, angiogenesis, and metastasis in liver cancer.^51^^,^^79^, ^80^, ^81^ Paracrine signaling involving the release of chemokine (C-C motif) ligand (CCL) 5, CXCL11, HGF, and follistatin like 1 (FSTL1) is exploited by CAFs to facilitate pulmonary metastasis in HCC.^51^^,^^79^^,^^82^^,^^83^ Various CAF-derived mediators, including CCL2, CCL7, CXCL16, IL-6, cartilage oligomeric matrix protein, heparin-binding endothelial growth factor (EGF)-like growth factor, and periostin, have been shown to encourage the invasion of HCC or CCA cells in coculture models, but their prometastatic roles have yet to be validated in vivo.^35^^,^^64^^,^^80^^,^^81^^,^^84^^,^^85^

### Chemotactic Cytokines

In 2022, Xu et al^79^ described a key role of CAF-secreted CCL5 in supporting HCC malignancy. Ablation of CCL5 or its cognate receptor CCR3/5 has been shown to impede CAF-driven cell aggressiveness and in vivo lung metastasis. Mechanistically, CCL5 stabilizes hypoxia-inducible factor 1 alpha (HIF1α) in HCC cells, which in turn increases zinc finger e-box binding homeobox 1 expression for EMT and hence induces metastasis.^79^ CXCL11 represents another key CAF-derived cytokine that fosters lung metastasis via paracrine activation of the circUBAP2/IFIT1/3 cascade.^51^ CXCL11 is enriched in metastatic HCC tissues, and its knockdown in CAFs weakens tumor migration in coculture and orthotopic models.^51^ miR-4756 was identified as a downstream factor that is repressed by CXCL11 and inactivates IFIT1/3, thus counteracting the migratory-promoting effect.^51^ Of note, circUBAP2 depletion reduces murine tumor migration and lung metastasis, whereas simultaneous blockade of miR-4756 antagonizes this suppression, indicating the significance of the CXCL11-circUBAP2/IFIT1/3 axis in HCC malignancy.^51^

### Growth Factors and Proteins

Early studies of liver myofibroblasts in CCA suggested that fibroblastic heparin-binding-EGF and periostin foster CCA cell invasion, potentially through endothelial growth factor receptor and ITGα5β1/PI3K/AKT pathways, respectively.^81^^,^^85^^,^^64^ In HCC, HGF is enriched in primary CAFs and can accelerate tumor progression and dissemination.^82^^,^^84^ HGF acts by stimulating the cMet/Erk1/2/FRA1/HEY1 axis in HCC cells, and fos-related antigen 1 (FRA1) silencing effectively retards HGF-induced tumor growth and metastasis.^82^ FSTL1 represents another CAF mediator with prometastatic ability, as illustrated by the elevated intrahepatic and lung metastasis in mice engrafted with HCC cells cultured in FSTL1-overexpressing CM and the reversed phenotypes in response to FSTL1 blockade.^83^ In vitro migratory and invasion assays verified these findings and showed that these aggressive features were enhanced by FSTL1 overexpression and repressed by its neutralization.^83^ Clinically, FSTL1 is primarily expressed in HCC CAFs and correlates with advanced cancer stage, reiterating its importance in HCC progression.^83^

## The Role of Cancer-Associated Fibroblasts in the Regulation of Cancer Stemness and Drug Resistance

Emerging work is revealing the stemness-driving capacities of CAFs in liver cancer. In fact, CAFs are a substantial source of cytokines and proteins that can promote cancer stemness, which is characterized by the gain of self-renewal and tumor-initiating abilities and drug resistance.^80^^,^^82^^,^^83^^,^^86^, ^87^, ^88^, ^89^, ^90^, ^91^ These CAF-derived mediators, along with the underlying stemness-driving mechanisms, have been extensively examined, some of which will be discussed next.

### Interleukin-6, Interleukin-33, and Cardiotrophin Like Cytokine Factor-1 Cytokines

CAF-derived IL-6 is suggested to play a key role in stemness induction in HCC in a signal transducer and activator of transcription 3 (STAT3)-dependent manner.^87^^,^^88^ Exogenous addition of IL-6 stimulated stem cell properties (marker gene expression and spheroid formation) and sorafenib resistance in HCC cells, whereas neutralizing IL-6 in CAF-CM or depleting hepatic STAT3 alleviated these effects.^87^^,^^88^ Recently, FAP^+^ CAFs from iCCA tumors were shown to secrete large amounts of IL-6 and IL-33 to induce stemness in an myeloid-derived suppressor cells (MDSC)-dependent manner.^91^ Specifically, these cytokines act on MDSCs by triggering STAT3-mediated expression and hyperactivation of 5-lipoxygenase, resulting in the release of leukotriene B4 mediator that supports iCCA cell stemness.^91^ Cardiotrophin-like cytokine factor (CLCF) 1 is also a stemness-stimulatory cytokine in HCC.^86^ In HCC cells, stemness marker gene levels and spheroid growth are supported by exogenous CLCF and repressed by fibroblastic CLCF1 or hepatic CLCF1 receptor (ciliary neurotrophic factor receptor [CNTFR]) silencing.^86^ Likewise, in a murine model, CLCF1/CNTFR depletion hampered tumorigenesis and the SRY-box transcription factor 2 score. Downstream CXCL6/E2F and TGFβ/MAPK signaling axes have been shown to mediate the stemness-enhancing effects, in which E2F transcriptionally controls stemness-associated gene expression.^86^

### Follistatin Like-1 and Hepatocyte Growth Factor

As discussed previously, FSTL1 and HGF independently promote HCC metastatic dissemination, a phenotype that can be attributed to stemness acquisition in cancer cells.^82^^,^^83^ Indeed, FSTL1 has been demonstrated to equip HCC cells with tumor-initiating and drug resistance abilities.^83^ FSTL1-overexpressing CM enhances self-renewal abilities and apoptotic resistance to sorafenib in HCC cells, and supports tumorigenicity in murine model and ex vivo culture of mice tumors.^83^ Consistently, FSTL1 blockade reverses these tumor-initiating phenotypes, sensitizes tumors to sorafenib, and prolongs mice survival.^83^ HGF is another major CAF mediator that elicits potent self-renewal abilities and chemoresistance in HCC cells, as indicated by increased spheroid formation and reduced apoptosis induced by cisplatin or doxorubicin in response to HGF administration.^82^ FRA1 acts downstream of HGF paracrine signaling in HCC cells, and its knockdown abrogates the HGF-induced stemness properties.^82^ Collaborating with these findings, another group showed that HGF could increase stemness marker expression, colony- and spheroid-forming capacities, and cisplatin and sorafenib resistance in HCC cells.^84^^,^^90^

## The Role of Cancer-Associated Fibroblasts in Nonalcoholic Steatohepatitis–Induced Hepatocellular Carcinoma

Nonalcoholic fatty liver disease (NAFLD) is the most common chronic liver disease worldwide; the global prevalence is around 20%–25% in adults.^92^ About 25% of patients with NAFLD progress to NASH, a more severe form of NAFLD characterized by steatosis-driven inflammation, hepatocellular injury, and liver fibrosis.^93^, ^94^, ^95^ NASH emerges as a key underlying risk factor of multiple end-stage liver cancer diseases, including cirrhosis, liver failure, and HCC.^94^^,^^96^ In fact, NASH has become the fastest growing cause of HCC according to a global epidemiology study from 2010 to 2019.^97^ Moreover, NASH might represent an important etiology accounting for failure to immunotherapies in HCC. In murine model, immune checkpoint inhibitor, anti-PD-1, failed to control tumor growth in NASH-related HCC; and in clinical studies, patients with NASH-HCC had worse prognosis and lower disease control rate on receiving immunotherapies compared with patients with other HCC aetiologies.^98^^,^^99^

In clinical and animal models of NASH, HSC is believed to be the origin of scar-forming myofibroblast. Single cell transcriptomic analysis of human NASH samples showed that injured hepatocytes stimulate myofibroblast differentiation and activation through TGFβ-1/2, Sonic hedgehog, and PDGFRBB signaling, which implicates the interplay between hepatocytes and activated fibroblasts in development of NASH-induced HCC.^100^ By examining the single cell expression profiles of HSCs extracted from a NASH model derived from *foz*/*foz* mice fed with western diet, heterogeneity of HSC was observed with 4 well-defined clusters with distinct functions.^101^ In patients with NASH-induced HCC, increased senescence and senescence-associated secretory phenotype were observed in CAFs, as evidenced by the increase in expression of α-SMA, p21, γ-H2AX, and IL-6. This senescence-associated secretory phenotype feature was correlated with higher incidence of NAFLD.^102^ Through analysis of gene expression omnibus database (GEO no. GDD3087), Myosin IC (MYO1C) expression was found to be upregulated in fibrotic liver of patients with NAFLD-induced HCC. Pharmacologic and genetic deletion of *Myo1c* attenuated TGFβ signaling of activated fibroblasts. *Myo1c*-knockout mice showed refractory to CCl_4_-induced liver fibrosis.^103^ Using 2 mouse NASH-induced fibrosis models, Asakawa et al. have analyzed the expression of CAFs by RNA sequencing analysis and showed that FGF9 was markedly upregulated in CAF, which was found to confer antiapoptotic and promigratory roles in hepatoma cells.^104^ In a stelic animal model NASH-HCC mouse model, a stepwise increase in HGF in plasma from the normal to the fibrosis and subsequent to HCC stage was demonstrated, which is in parallel with accumulation of activated fibroblasts.^82^ Collectively, these molecules might represent novel targets for alleviating liver fibrosis or delaying the clinical progression from NASH to HCC; but the actual therapeutic benefits in NASH-induced HCC require further investigations.

## Crosstalk Between Cancer-Associated Fibroblasts and Immune Cells in the Tumor Microenvironment

CAFs can shape the immune microenvironment toward a tolerant milieu through extensive crosstalk with various immune cell types. An early in vitro study illustrated that CAFs deactivate natural killer (NK) cells and impair their cytotoxicity against HCC cells by secreting prostaglandin E2 (PGE2) and indoleamine 2,3-dioxygenase (IDO), and interestingly, the release of these factors depends on the presence of NK cells, implying plausible crosstalk between CAFs and NK cells in the HCC TME.^56^ Other coculture studies demonstrated that CAFs secreted SDF1 alpha to recruit neutrophils, monocytes, or dendritic cells by interacting with their surface C-X-C chemokine receptor type (CXCR) 4.^105^, ^106^, ^107^ IL-6 is additionally released from CAFs and stimulates the JAK/STAT3 pathway in these immune cells, potentiating their differentiation into immunosuppressive subtypes, which in turn represses T-cell proliferation and antitumor activity.^105^, ^106^, ^107^ These early in vitro studies provided insights into the immunomodulatory roles of CAFs in liver cancer.

In 2016, Yang et al^108^ provided the first in vivo evidence, to our knowledge, showing that CAFs promoted HCC tumor growth by attracting immunosuppressive cells. Using a coimplantation model, the researchers revealed that FAP^+^ CAFs were indispensable for fostering MDSC recruitment and tumor growth, and importantly, FAP overexpression was sufficient to equip normal fibroblasts with tumor-promoting abilities.^108^ Mechanistically, FAP stimulated uPAR/FAK/Src/JAK2 autocrine signaling in CAFs and activated STAT3, inducing the expression and release of CCL2, which in turn recruited MDSCs in a CCR2-dependent manner.^108^ Depleting fibroblastic STAT3 or CCL2, or murine CCR2 attenuated the FAP-induced MDSC infiltration and tumor-promoting effects in vivo.^108^ Extending this work, the group later described that the MDSC-recruiting and protumorigenic features of FAP/STAT3/CCL2 signaling were conserved in iCCA, reiterating the importance of CAFs and FAP signaling in generating an immunosuppressive stroma that is permissive for tumor progression.^109^ Apart from MDSCs, CAFs also exert immunosuppressive effects on macrophages via the endosialin/GAS6 axis in HCC.^110^ Endosialin, a transmembrane protein highly expressed in HCC CAFs, has been revealed to enhance tumor growth and macrophage infiltration in vivo by interacting with its surface CD68.^110^^,^^111^ And M2 polarization of macrophages is achieved through GAS6 release.^110^ To target this CAF-macrophage crosstalk, the group generated an anti-endosialin human antibody (IgG78) that tags glycosylated endosialin for lysosomal degradation, which was shown to successfully deplete CAF-induced M2 infiltration and tumorigenesis in subcutaneous and orthotopic xenografts, verifying the immunoregulatory and protumoral abilities of endosialin and rendering IgG78 an appealing therapeutic candidate.^110^

Recently, Song et al^86^ elucidated an intricate crosstalk among CAFs, HCC cells, and tumor-associated neutrophils triggered by CAF-derived CLCF1 cytokines. The interaction of CLCF1 with its receptor on HCC cells (CNTFR) contributes to N2 neutrophil infiltration and tumor propagation.^86^ Using a murine model engrafting CAFs-HCC cells followed by tail-vein infusion of human neutrophils, they showed that CLCF1 or CNTFR depletion abrogated neutrophil recruitment and tumor proangiogenic growth triggered by CAFs.^86^ CLCF1/CNTFR induction in HCC cells activates the release of CXCL6 and TGF-β, which function to attract neutrophils via CXCR1/2, and polarize them to the N2 protumorigenic subtype, respectively.^86^ It is noteworthy that ERK1/2 seems to be a key regulator of the CLCF1-CXCL6/TGFβ axis. In HCC cells, ERK1/2 acts downstream of CNTFR induction and controls CXCL6 and TGFβ secretion. Meanwhile, in CAFs, it is activated by CXCL6 and TGFβ to reinforce CLCF1 release, forming a positive feedback loop to foster HCC progression. ERK1/2 blockade reverses N2 phenotype in vitro and in vivo and mitigates the protumorigenic effects imposed by CLCF1.^86^

Collectively, CAFs promote immunosuppression by direct or indirect crosstalk with immune cells and educating them to harbor tolerant traits. Several key axes, including SDF1a-CXCR4, FAP/STAT3/CCL2-CCR2, Endosialin-CD68, and CLCF1-CNTFR/CXCL6, are deployed by CAFs for immune cell recruitment.^86^^,^^105^, ^106^, ^107^, ^108^, ^109^, ^110^ Notably, CLCF1 and endosialin are primarily expressed on HCC CAFs and associated with poor clinical outcome in HCC, whereas heightened expression of FAP predicts poor prognosis in iCCA patients, suggesting the clinical relevance of these signaling cascades.^86^^,^^109^^,^^110^ As shown by coculture studies, CAF-derived soluble factors (IL-6, PGE2, and IDO) seems to induce suppressive phenotypes in NK cells, neutrophils, monocyte, or dendritic cells*.*^58^^,^^105^, ^106^, ^107^ In murine models*,* CAF-derived GAS6, TGF-β, and CCL2 induce immunosuppressive M2 and N2 differentiation and MDSC recruitment, thereby augmenting HCC propagation or metastasis.^86^^,^^108^, ^109^, ^110^

## Therapeutic Implications

As described in previous sections, CAFs drive immunosuppression, stemness, and metastasis in liver cancer. These protumorigenic features, along with their abundance in the TME and genetic stability, render them attractive targets for anticancer therapies.^112^ Therapeutic approaches against CAFs have been extensively reviewed.^52^^,^^113^, ^114^, ^115^ Here, we briefly discuss the recent advancements in CAF-targeting therapies, particularly those applicable to liver cancer.

### Cancer-Associated Fibroblasts Elimination

In the last decade, studies have engrossed on diminishing the prominent FAP^+^ CAFs subset to control tumorigenesis. DNA vaccines, CAR-T-cell adoptive transfer, and oncolytic virus-based approaches deplete FAP^+^ CAFs and restrain the tumor growth of mesothelioma, and colon, pancreatic, and lung cancers in murine models.^116^, ^117^, ^118^, ^119^ Of note, a research group generated a novel FAP immunogen that synergies with antitumor antigen vaccines in eliciting antitumor immunity, thus attenuating lung and prostate tumoral growth with increased mice survival.^120^ Nevertheless, the applicability of these approaches might be limited because of the widespread expression of FAP, especially in bone marrow and muscle, and systemic targeting of FAP^+^ cells might result in cachexia, bone toxicity, muscle loss, or even death.^121^^,^^122^ To avoid systemic toxicity, recent studies have focused on developing strategies that can specifically eradicate FAP^+^ CAFs.^123^^,^^124^ Zhen et al^123^ developed a nanoparticle-based photoimmunotherapy that enables selective elimination of FAP^+^ CAFs, which efficaciously retarded tumor growth without endangering other FAP^+^ populations. Another approach involves the expression of FAP-bispecific T-cell engagers (FAP-BiTE), a bispecific antibody directed against fibroblastic FAP and CD3ε on T cells using the adenoviral major late promoter system.^124^ This system allows tumor-restricted expression of FAP-BiTE, which elicits potent T-cell cytotoxicity against CAFs in human biopsies (malignant ascites and prostate cancer tissue).^124^ In fact, in HCC, mere expression of FAP can imbue normal fibroblasts with a protumorigenic niche.^108^ Moreover, FAP autocrine signaling in CAFs recruits MDSCs (via CCL2) and endows MDSCs with stemness-enhancing abilities (via IL-6/33), thus augmenting propagation and initiation of HCC or iCCA tumors.^108^^,^^109^ As such, FAP-targeting approaches might offer therapeutic benefits and seem warranted in liver cancer.

### Targeting Cancer-Associated Fibroblasts Precursors or Secretome

Methods to suppress CAF functions are also in development for combating liver tumorigenesis. One way is to prevent the conversion from precursor cells to CAFs. Metformin and curcumin were recently illustrated to inactivate HSCs, the most common CAF precursor in liver cancer.^125^^,^^126^ Metformin, a commonly used antidiabetic drugs, can impede murine tumorigenesis in a liver fibrosis model.^125^ Natural compound curcumin can suppress EMT and alleviate cell aggressiveness induced by HSCs through abrogating the HIF-1α/CTGF axis in HCC cells.^126^ Peritumoral fibroblasts represent another source of CAFs in HCC and are capable of supporting tumor growth in vivo.^44^ LPA is released from HCC cells that foster peritumoral fibroblasts transdifferentiation, and using an LPA inhibitor (α-bromomethylene phosphonate [BrP]-LPA) can readily block the transdifferentiation and slow tumorigenesis.^44^

Another way to impair CAF function is to ablate their paracrine secretion. FSTL1 drives stemness acquisition and malignant progression in HCC cells, patient-derived organoids, and tumors, and effective tumor control is accomplished by FSTL1 blockade.^83^ Critically, FSTL1 is tightly linked to sorafenib resistance, and combining an anti-FSTL1 antibody with sorafenib prolonged mice survival with reduced tumor stemness, illustrating the potential clinical benefits of FSTL1 targeting, either as a single agent or in combination with sorafenib.^83^ Some other mediators, CCL5 and CXCL11, confer HCC cells’ ability to disseminate to lung.^51^^,^^79^ Abrogation of these mediators, their interacting receptor (CCR3/5), or downstream signaling molecules (HIF1α, circUBAP2) ameliorates tumor malignancy, and thereby representing actional targets for HCC treatment.^51^^,^^79^ In addition to soluble factors, CAFs also secrete exosomes to the TME. miR-320a is found to exhibit antitumor properties, and its expression is reduced in CAF-derived exosomes.^113^ Elevating the level of miR-320a by overexpression in CAFs or exogenous addition to HCC cells restores antitumoral effects and prevents metastasis, suggesting its use in HCC treatment.^127^

### Targeting Cancer-Associated Fibroblasts–Mediated Crosstalk

In tumor stroma, CAFs actively communicate with immune cells to elicit their protumorigenic capacities. Weakening CAF-immune cells crosstalk prevents CAF-driven tumor growth, thus representing an alternative modality for cancer treatment. As discussed previously, in liver cancer, CAFs crosstalk with immune cells through CCL2, CLCF1, or endosialin release, favoring tumoral infiltration of MDSCs, N2 neutrophils, or M2 macrophages with resultant tumor propagation.^86^^,^^108^^,^^109^ Depleting these mediators or receptors on respective immune cells (ie, CCR2 and CNTFR) can reignite antitumor immunity and constrain tumor growth, rendering these factors attractive therapeutic targets.^86^^,^^108^^,^^109^ Furthermore, IL-6, an abundant CAF-secreted cytokine, has been documented to indirectly crosstalk with T cells via PD-1/PD-L1 signaling, hence restricting their proliferation and antitumor immunity in HCC.^105^ Consistent with this finding, combinational treatment involving IL-6 and PD-L1 neutralization effectively controlled tumor size and improved survival in HCC mouse model, suggestive of plausible targeting of this CAF-T-cell crosstalk in liver cancer.^128^

TGFβ signaling represents an important cascade that mediates crosstalk of CAFs, and the possibilities of therapeutically targeting this factor have been extensively investigated. In HCC, TGFβ signaling mediates CAF-cancer cell crosstalk to control tumor phenotype.^83^ TGFβ is emanate from both CAFs and HCC cells, acting as autocrine and paracrine factors to stimulate FSTL1 signaling in CAFs, eventually leading to increased HCC stemness and metastatic dissemination.^83^ In fact, a prior phase II clinical trial has investigated the use of the TGFβ inhibitor, galunisertib, in patients with advanced HCC and revealed an improved prognosis in TGFβ responders.^129^ However, fibroblastic TGFβ signaling is also implicated in immune evasion in a reconstituted colon cancer model with liver metastases.^116^ Coadministration of galunisertib and anti-PD-L1 antibodies elicited a potent Tc antitumor response, attenuating most metastases and prolonging mice survival.^130^ A similar finding was reported in another study using antibodies against TGFβ and PD-L1, which inactivated the fibroblastic TGFβ pathway and profoundly inhibited tumor growth in murine mammary and colorectal carcinoma models.^131^ These preclinical studies further support the notion that CAF-mediated crosstalk controls tumorigenesis, and their disruption might offer therapeutic value. Additionally, although combination modalities involving in CAF-targeting and anti-PD-L1 drugs deemed effective in other cancers, it is crucial to investigate whether or not CAF-directed agents can overcome anti-PD-L1 resistance and yield clinical benefits in patients with liver cancer.

## Conclusions and Future Perspectives

**Figure 2:**
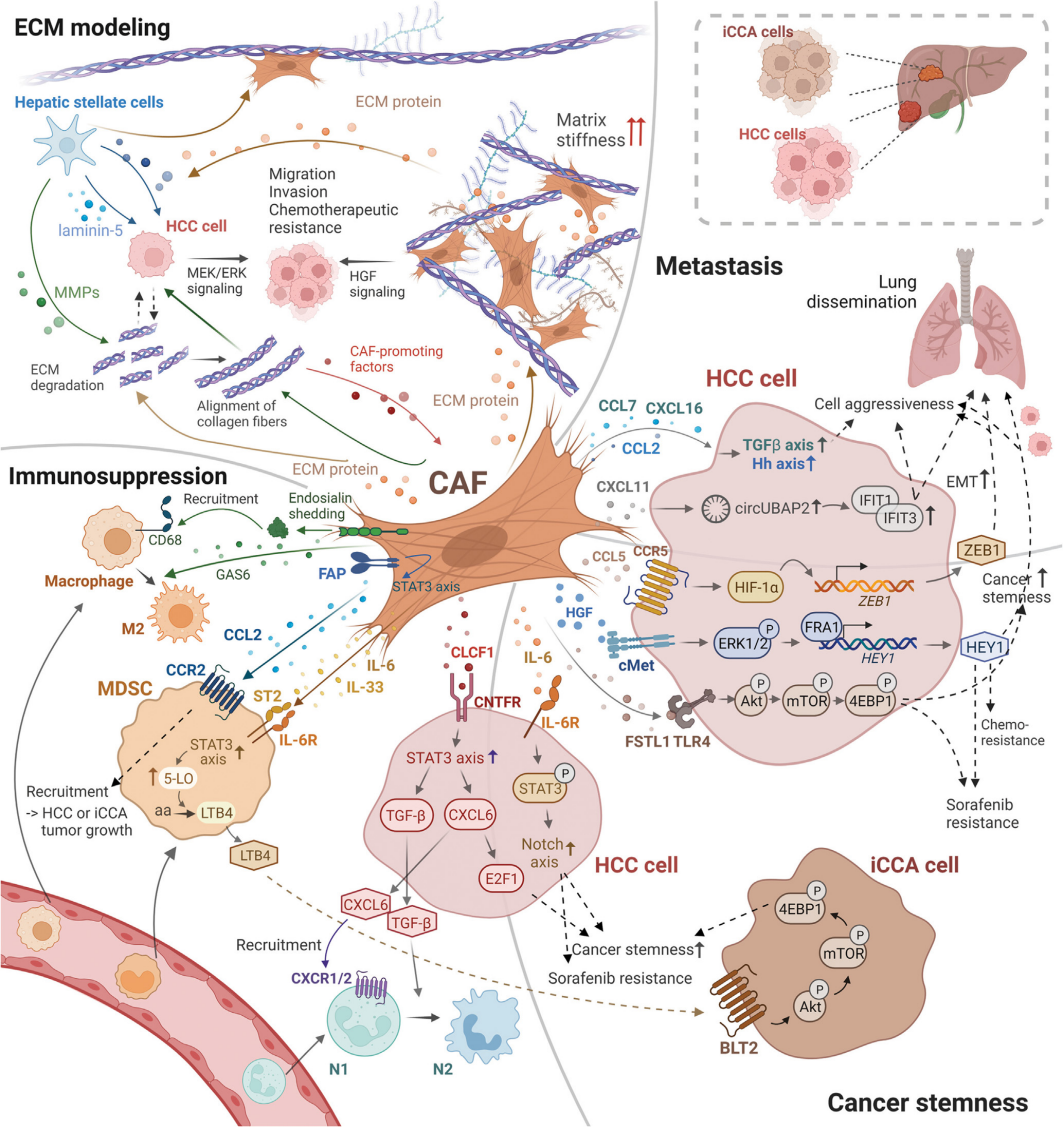
**CAFs and their secretome remodel the stromal environment and support immune evasion, metastasis, and stemness acquisition of PLC.** Secretory functions of CAFs regulate ECM remodeling (*top left*); tumor immunity (*bottom left*); metastatic dissemination (*top right*); and self-renewal, tumor-initiating, and drug resistance capacities (*bottom right*) of PLC cells. CAFs secrete ECM proteins that contribute to matrix degradation, deposition, and stiffness (*top left*). ECM protein is also required to drive HSCs transdifferentiate into CAFs. ECM degradation and alignment of collagen fiber as alternative mechanisms in ECM remodeling could help to deliver CAF-promoting factors and results in CAFs activation. As a feedback loop, the activated CAFs could guide the cancer cells via alignment of collagen fiber for migration. In PLC TME, CAFs release various cytokines to tumor stroma, directly or indirectly foster immune cell infiltration, and polarize them to inhibitory subtypes, thus generating an environment favorable for tumor propagation (*bottom left*). CAF secretome also actively engages in HCC aggressive behaviors and metastatic colonization in lung, predominantly by inducing stemness features in HCC cells (*top and bottom right*). CAF mediators directly enhance renewal, tumorigenic, and therapeutic resistance properties in HCC cells, and indirectly promote these characteristics in iCCA cells in an MDSC-dependent manner (*bottom right*).

Taken together, accumulating evidence supports the tumor-promoting role of CAFs in PLC via their direct effects on cancer cells or indirectly via other cell types within the TME. In this review, we summarized the roles of CAFs in ECM remodeling, metastasis, cancer stemness, and the induction of an immunosuppressive microenvironment (Figure 2). It is critical to further investigate the mechanisms involved in CAF-induced tumor progression to identify new therapeutic targets for PLC treatment. Notably, it is critical to determine the crosstalk between CAFs and other immune cells to increase the current response rate of immune checkpoint therapies. For a better understanding of CAF biology in PLC, it is crucial to identify the origin of CAFs in PLC. With the development of scRNA sequencing, several origins of CAFs in PLC have been proposed, including HSCs, PFs, fibrocytes, mesothelial cells, and MSC. Investigations are currently underway to evaluate the therapeutic implications of blocking the transition of these precursor cells to CAFs. Among these studies, CAF heterogeneity exists within PLC. The identification of different subsets of CAFs will provide insight into the development of accurate and efficient strategies against CAFs. However, the identification of subsets of CAFs in PLC requires fresh tumor tissues, which may result in bias in cell preparation with the loss of rare cell populations. With the advent of single-nucleus RNA sequencing, researchers have been able to better identify the subsets of CAFs and their interactions with immune cells in frozen tissues. In addition, scRNA sequencing identified specific subsets of CAFs based on transcriptional gene expression signatures, but this analysis does not represent the protein level. To overcome this limitation, scRNA or single-nucleus RNA sequencing analysis coupled with spatial transcriptomics will provide a better resolution of CAF subsets, and their crosstalk with microenvironment components in PLC. Because of the existence of CAF heterogenicity, the markers of CAFs are also heterogeneous. To precisely target CAF populations, the current research direction is focused on identifying specific CAF markers without hampering normal tissues. Regarding the functional studies of CAFs in PLC, researchers have relied on in vitro coculture systems that have evolved from simple 2-dimensional monocultures to 3-dimensional systems with CAFs derived from mouse and primary PLC patients in the past decade. Recently, increasing efforts have been made to understand CAF biology to elucidate the complex interaction among cancer cells, CAFs, and other immune cells using organoid cultures that closely mimic the in vivo TME. To improve the viability of cells and maintain biologic activity in this coculture system, researchers have used hyaluronan-gelatine hydrogel as a matrix for potential in vitro drug testing.^132^ Apart from this, in vivo marker-based CAF-specific transgenic mouse models and patient-derived xenograft models are also used in CAF research. With a better understanding of CAF biology in the TME, we believe that new therapeutic strategies against CAFs for PLC therapy lies ahead.
